# Antroduodenal motility in neurologically handicapped children with feeding intolerance

**DOI:** 10.1186/1471-230X-4-19

**Published:** 2004-09-01

**Authors:** Steven L Werlin

**Affiliations:** 1 Department of Pediatrics, The Medical College of Wisconsin and The Children's Hospital of Wisconsin Milwaukee, WI United States of America

## Abstract

**Background:**

Dysphagia and feeding intolerance are common in neurologically handicapped children. The aim is to determine the etiologies of feeding intolerance in neurologically handicapped children who are intolerant of tube feedings.

**Methods:**

Eighteen neurologically handicapped children, followed in the Tube Feeding Clinic at the Children's Hospital of Wisconsin who were intolerant of gastrostomy feedings. The charts of these 18 patients were reviewed. Past medical history, diagnoses, history of fundoplication and results of various tests of gastrointestinal function including barium contrast radiography, endoscopy and antroduodenal manometry were documented.

**Results:**

Five of 11 children had abnormal barium upper gastrointestinal series. Seven of 14 had abnormal liquid phase gastric emptying tests. Two of 16 had esophagitis on endoscopy. All 18 children had abnormal antroduodenal motility.

**Conclusions:**

In neurologically handicapped children foregut dysmotility may be more common than is generally recognized and can explain many of the upper gastrointestinal symptoms in neurologically handicapped children.

## Backround

Oral pharyngeal dysphagia due to disordered swallowing has become increasingly recognized in children with cerebral palsy and other neurodevelopmental disorders. This has led to the increasing use of enteral tube feedings either for full or supplemental nutritional support. Symptoms of foregut dysmotility, such as vomiting, retching gagging and bloating, are often associated with tube feeding in neurologically handicapped children [1-4]. Previous studies have demonstrated that recurrent vomiting, aspiration and/or failure to thrive may be present in as many as 10–15% of institutionalized patients with psychomotor retardation. Antroduodenal motor function has been little studied in such children [5,6].

In order to elucidate the mechanisms behind these symptoms we reviewed the charts of a group of children followed in the Tube Feeding Clinic at the Children's Hospital of Wisconsin with neurological dysfunction, who were intolerant of tube feedings and who had undergone antroduodenal motility studies as part of their evaluations.

## Methods

The charts of 18 neurologically handicapped children (mean age 4 years, range 1–10 years; 10 males) with dysphagia and symptoms of foregut motility were reviewed. The symptoms, underlying disorders, feeding route, the presence of fundoplication, and the use of prokinetic agents and H_2 _b1receptor antagonists are summarized in Table 1. All except one patient were completely or partially fed enterally. One patient ate orally but required frequent venting of his gastrostomy.

**Table 1:** Patient population

**Patient Number**	**Age (y)**	**Diagnosis**	**Symptoms**	**Feeding Route**	**UGI**	**Gastric Emptying**	**EGD**	**PEG**	**Fundoplication**	**Other**	**Medications**	
											**H2RA**	**Cisapride**
1	9	CP GER	vomiting retching	oral	paraesophageal hernia	normal	normal		+	gastric bezoar		
2	4	feeding aversion	vomiting retching	gastrostomy	esophageal dysmotility	ND	esophagitis	+			+	+
3	3	Down's syndrome	gagging retching	gastrostomy	normal	delayed	normal		+			
4	2	cerebral dysgenesis seizures	Irritability	jejunostomy	GER	delayed	esophagitis	+				omeprazole
5	5	chromosome 19 deletion, GER subglotic stenosis	retching	gastrostomy	normal	normal	ND		+			
6	4	hydrocephalus	retching	gastrostomy	normal	normal	normal	+	+		+	
7	1	CP	irritability	gastrostomy	paraesophageal hernia	normal	normal		+		+	+
8	2 1/2	cerebral atrophy recurrent aspiration pneumonia	vomiting	gastrostomy	GER	normal	normal	+			+	+
9	2	diphragmatic hernia GER	vomiting	jejunostomy	GER	delayed	ND	+		surgical jejunostomy	+	+
10	5	CP spina bifida	retching bloating constipation	jejunostomy	normal	normal	normal		+	surgical jejunostomy	+	+
11	9	Floating Harbour syndrome	retained food vomiting	oral	ND	rapid	retained food	+	+		+	+
12	17	mitochondrial disease	vomiting diarrhea retching gagging bloating	gastrostomy	esophageal dysmotility	delayed	normal		+		+	+
13	3	CP GER	retching gagging	gastrostomy	ND	normal	normal		+			+
14	6	charge syndrome chromosome 13 Deletion	retching gagging	gastrostomy	normal	normal	esophagitis		+		+	+
15	2	CP hepatoblasoma	vomiting	gastrostomy	paraesophageal hernia	delayed	normal		+	Nissen breakdown liver resection pyloroplasty	+	+
16	1	CP	vomiting	gastrostomy	GER	delayed	esophagitis	surgical gastrostomy	-	pyloroplasty	+	+
17	2	feeding aversion	vomiting	gastrostomy	normal	+	normal	+			+	+
18	10	CP seizures hydrocephalus	vomiting retching gagging bloating	TPN	+	+			+		+	+

GER: gastroesophageal reflux CP: cerebral palsy: NE: not done TPN: total parenteral nutiriton

Following an overnight fast, antroduodenal motility studies were performed using a multilumen catheter with 8 recording ports spaced 2.5–5 cm apart, passed through the gastrostomy either under fluoroscopic guidance or endoscopically, connected to a low compliance, pneumohydraulic capillary infusion system (Arndorfer Medical Specialties, Greendale, WI) and a computerized motility system (Redtech, Calabasas, CA). Fasting activity was recorded for 3–4 hours. Erythromycin (1 mg/kg) was given intravenously over 10 minutes and the recording continued for another hour. Octreotide (0.5 mcg/kg) was then given intravenously over 5 minutes. 45 minutes later a liquid meal was given followed by an additional 2–3 hour recording period. The meal varied and consisted of the usual formula and volume given at home. Patients receiving jejunal feedings or TPN were given a bolus gastrostomy feeding. In the one patient receiving TPN, the TPN was discontinued during the study. Prokinetics were stopped at least 48 hours prior to study.

Phase 1 of the MMC was defined as motor quiescence. Phase 2 was defined as the time between Phases 1 and 3 and is characterized by random contractions of varied amplitude and frequency. Phase 3 of the MMC is characterized by an aborally propagating cluster of repetitive contractions with a frequency of 11–13/minute in the duodenum and 3/minute in the antrum with a duration of 3–10 minutes. The tracings were analyzed by visual inspection.

This study was approved by the Research and Publications Committee/Human Rights Review Board of the Children's Hospital of Wisconsin and the Institutional Review Board of the Medical College of Wisconsin.

## Results

Eleven children had had recent upper GI series. Of these 5 were normal, 3 had gastroesophageal reflux and 3 had paraesophageal hernias (all following fundoplications), 1 had a bezoar and 1 had esophageal dysmotility. Fourteen patients had liquid phase gastric emptying studies. Of these 7 were normal, 6 had delayed emptying and 1 had rapid emptying. Two of 16 patients who had had recent endoscopies had esophagitis, 14 were normal. The diagnoses and clinical histories of the patients are summarized in Table 1. Twelve of the 18 patients had had a fundoplication and 9 of the 12 had had a pyloroplasty. Indications for fundoplication were frequently poorly described in the medical records but typically included vomiting and feeding intolerance. The incidence of symptoms such as retching and sweating could not be determined

**Figure 1:**
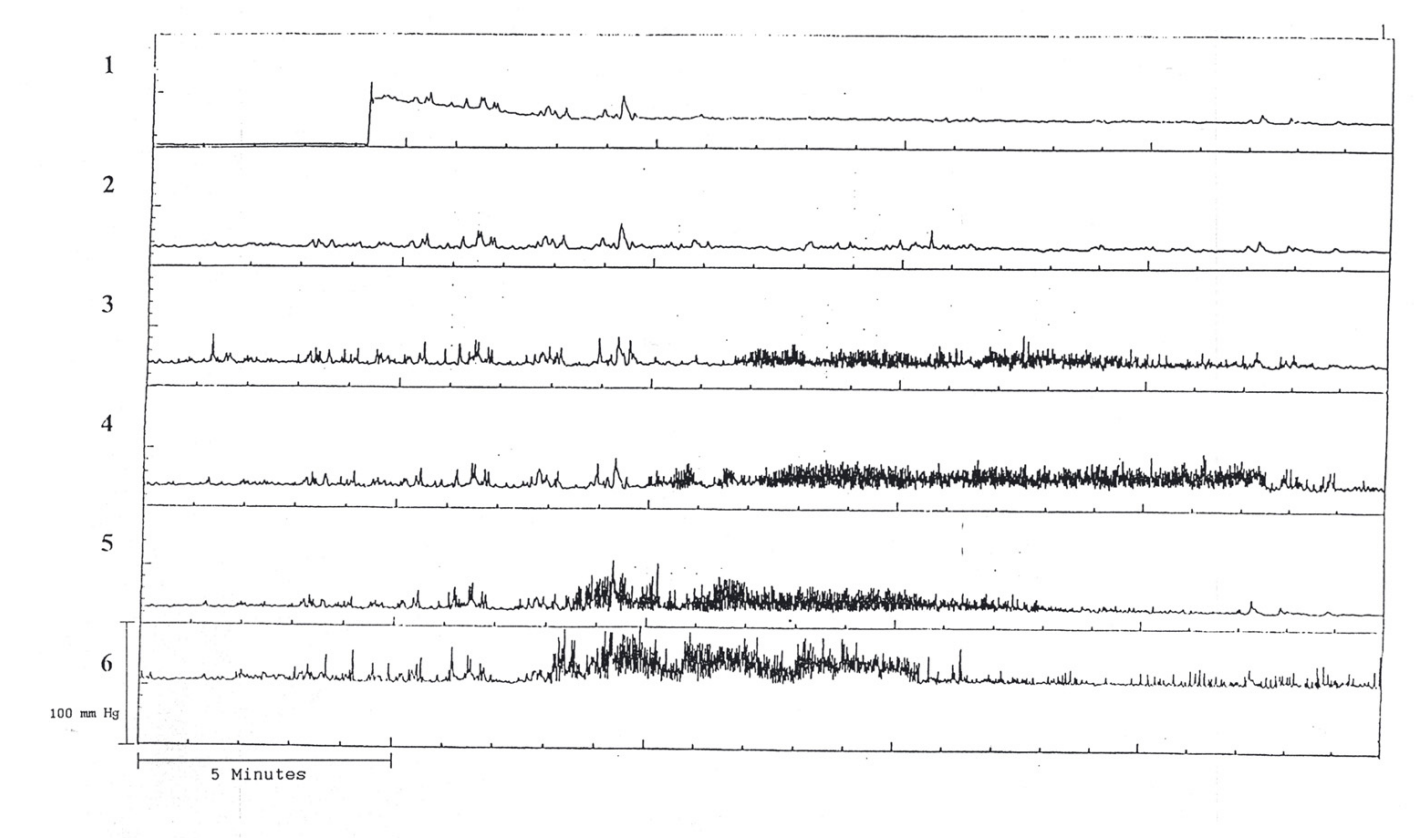
An 8-year-old boy who was TPN dependent demonstrates reverse peristalsis. Note lack of antral contractions. Channel 1–2 antrum; 3–6 duodenum.

**Figure 2:**
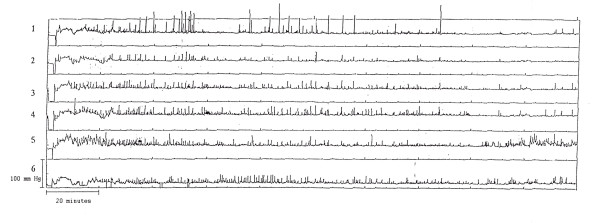
Lack of fasting phase 3 activity during a 3 hour monitoring period in an 8-year-old boy. He also had no phase 3 like activity following erythromycin. Channel 1 antrum; 2–6 duodenum.

**Figure 3:**
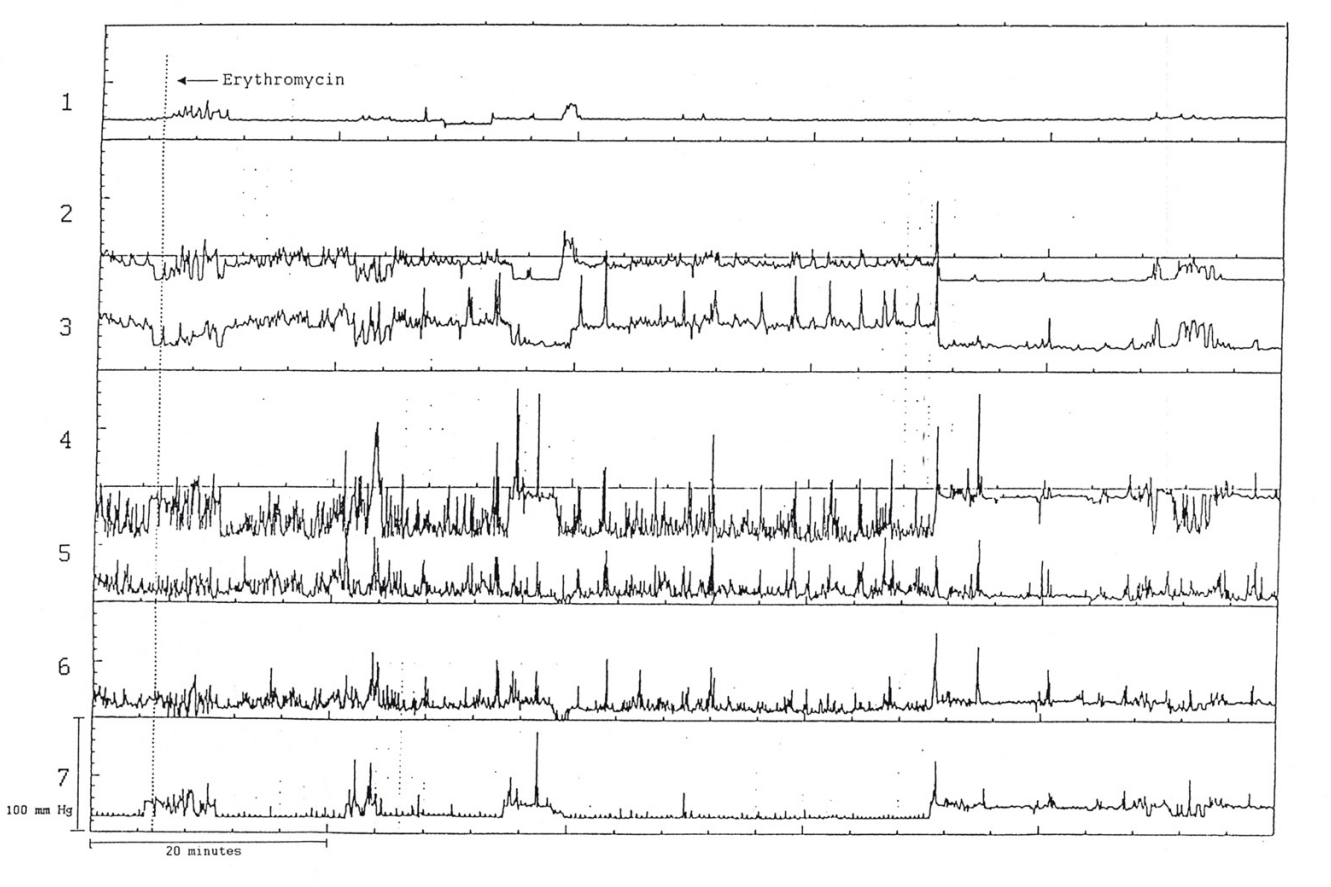
Lack of responsiveness to erythromycin in a 3-year-old boy with feeding aversion. This patient also had postprandial hypomotility. Channels 1 stomach; 2–3 antrum; 4–7 duodenum.

No patients had a normal antroduodenal motility study (Figures 1,2,3). In the fasting state 12/18 failed to have phase 3 of the MMC. Eight had predominantly phase 2 activity and did not demonstrate normal fasting phase 1. Nine had non-propagating clusters. One had MMCs that propagated in a retrograde fashion.

Following erythromycin eight had a normal response consisting of antral contractions with a frequency of 3/min followed by phase 3 like activity in the duodenum. Three patients had no response and seen had abnormal responses consisting of abnormal clusters in three, no antral response in three and no duodenal response in three.

Fourteen patients had a normal response to octreotide consisting of cessation of antral activity and the development of phase 3 activity in the duodenum. Two patients had continued antral contractions and two did not develop phase 3 duodenal activity. These patients had non-propagating duodenal clusters.

Fourteen patients had normal postprandial phase 2-like activity. Eight developed premature phase 3 activity within 30 minutes following the meal. Seven patients failed to develop phase 3 activity during the 2–3 hour postprandial monitoring period and seven had no antral contractions in the postprandial monitoring period. One patient had a retrograde MMC.

Three patients developed severe pain or irritability associated with antral or duodenal contractions following erythromycin [2] or octreotide [1]. There was no correlation between any constellation of symptoms and manometric abnormalities.

## Conclusions

Foregut dysmotility is common in children with neurodevelopmental disorders such as cerebral palsy [1-4]. Up to 75% of institutionalized children with psychomotor retardation have GER [7-11]. A number of investigators have reported that neurologically handicapped children have abnormalities of lower esophageal function [12,13]. Delayed gastric emptying is common in such patients [1,2]. Many of these patients undergo fundoplication. Continued symptoms of GER following fundoplication or the development of new symptoms such as retching and gagging suggests that a more generalized foregut motility disorder is present in many of these patients [5]. The rates of complications of surgical treatment of GER that might relate to foregut dysmotility include breakdown of the wrap (0.9–13%) and the gas bloat syndrome (1.9–8%) [14]. Other complications not reported in enough detail to estimate complication rates include dumping, and gastroparesis.

Ravelli and Milla have shown that gastric electrical activity as measured by the electrogastrogram (EGG) was abnormal in 31/50 neurologically handicapped children [2]. Eleven of 18 patients who were symptomatic after fundoplication had gastric dysrhythmias. Richards et al showed that neurologically impaired children with pallor, sweating, retching or forceful vomiting preoperatively were at high risk for postoperative retching and vomiting. They hypothesized that these symptoms were indicative of activation of the emetic reflex and that children with these symptoms had a more generalized disorder than those children without such symptoms [15].

In a previous study we compared gastric electrical activity as measured by EGG in a group of neurologically handicapped children who were tolerant of their tube feedings to a group that were intolerant or symptomatic during tube feedings [16]. The percentage of children in each group who had undergone fundoplication was the same. We found that although the percentage of time that normogastria, bradygastria and tachygastria were present was not different in the 2 groups, there was a significant difference in the postprandial power between the groups. This finding suggests that symptoms present in these patients such as vomiting, retching and gagging might be due to an underlying foregut motor disorder.

There have been few reports of antroduodenal motility, which have focused on neurologically handicapped children with feeding intolerance. DiLorenzo and colleagues reported that 25/28 children who remained symptomatic following fundoplication had abnormal antroduodenal motility [5]. Similar to our patients a wide variety of abnormalities were found. These authors did not report how many of their patients that had neurological handicaps. Miki et al found that fasting antroduodenal motility was abnormal in 11 neurologically impaired children with symptoms of gastroesophageal reflux [6].

Due to the nature of our patients there are some limitations in the design and interpretation of our study. Because many of our patients had had multiple formula changes, we decided to use the formula, which the child was receiving at the time of the study, so that any symptoms occurring during the studies could not be attributed to a formula change. Bolus feeds were given to all patients during the study even those who had been receiving drip feeds. The fasting state could not be recorded for 4 hours in all patients, thus it is possible that absence of phase 3 of the MMC in these patients might not be abnormal. Since normal manometry data have not been published for children, non propagating clusters may or may not be normal in our patients.

Since only 12 of our 18 patients had undergone fundoplication, we agree with DiLorenzo et al [5] that these motor abnormalities were not caused by surgery, rather we believe that the underlying motility disorder was more generalized than had been recognized at the time of fundoplication.

In this study we have confirmed that the incidence of foregut dysmotility is very high in neurologically handicapped children with feeding intolerance. Prokinetics and acid suppression did not resolve the symptoms in our patients. Twelve of our 18 patients had had fundoplications and two had undergone two fundoplications in unsuccessful attempts to control what had been thought to be reflux symptoms. While there is no way to know how much abnormal antroduodenal motility contributed to our patient's feeding disorders, following antroduodenal manometry a number of our patients were treated successfully with jejunal feeding, suggesting that while they had foregut dysmotility, midgut motility is normal.

In neurologically handicapped children foregut dysmotility may be more common than is generally recognized and can explain many of the upper gastrointestinal symptoms in neurologically handicapped children. Thus in this patient population generalized foregut dysmotility may mimic reflux and the decision to perform a fundoplication should be made very cautiously and only after a complete evaluation of foregut motility particularly in children with gagging retching and forceful vomiting.

## Competing Interests

None declared.

## Pre-publication history

The pre-publication history for this paper can be accessed here:


